# Beyond the gift: donor motivations and family experiences as drivers of body donation programs

**DOI:** 10.3389/fpubh.2025.1720025

**Published:** 2026-01-05

**Authors:** Valérie Defaweux, Olivier Prygiel, Louis Schieres, David Mutombo Mwembo, Anne-Marie Etienne, Allyson Fries, Alain Botte, Caterina Marchese, Murielle Wouters, Pierre Bonnet, Marc Radermecker, Aude Lagier

**Affiliations:** 1 Human Anatomy Laboratory, Department of Biomedical and Preclinical Sciences, Faculty of Medicine, University of Liège, Liège, Belgium; 2 Department of Psychology, Health Psychology, Faculty of Psychology, Speech Therapy and Educational Sciences, University of Liège, Liège, Belgium; 3 Laboratory of Human Histology, Department of Biomedical and Preclinical Sciences, Faculty of Medicine, University of Liège, Liège, Belgium

**Keywords:** anatomical education, behavior change wheel, bereavement, body donation, donor motivations, family perspectives, public health promotion

## Abstract

**Background:**

Body donation to science provides indispensable resources for medical education and research, yet shortages remain widespread. Understanding the interplay between donor motivations and family experiences is essential to sustain donation programs and to position them as public health education initiatives.

**Methods:**

We conducted *a mixed-methods exploratory study* at the University of Liège (Belgium), combining self-administered questionnaires from registered donors (*n* = 104) with surveys of donor families (*n* = 10). Quantitative data were analyzed descriptively and with nonparametric tests, while qualitative responses underwent thematic content analysis. The Behavior Change Wheel (BCW), the Protection Motivation Theory (PMT), and prosociality frameworks guided interpretation.

**Results:**

Scientific utility was the leading motivation (56.7%), followed by symbolic meaning, altruism, and gratitude. Nearly all donors informed their relatives (96.2%), whose reactions varied by occupational category. Families expressed overall satisfaction but frequently described a “double bereavement” at death and at restitution. Word-of-mouth was the predominant channel of information (52.9%), though many respondents called for broader outreach. Within the PMT framework, donation was driven by high perceived response efficacy, reinforced self-efficacy (accessible information, family dialogue), and limited but salient emotional costs. Prosocial and altruistic factors (empathy, responsibility, symbolic legacy) complemented protective motivations, framing donation as a costly yet meaningful prosocial act.

**Discussion:**

Integrating PMT and prosociality provides a novel model to explain why donation simultaneously addresses a perceived systemic shortage (protective motive) and a desire to contribute to the common good (altruistic motive). Family support functions as a key moderator, amplifying motivation and reducing perceived costs. Within the BCW, interventions should enhance capability (clear communication, bereavement support), expand opportunity (media campaigns, standardized rituals, faculty presence), and sustain motivation (student/educator testimonials, family recognition).

**Conclusion:**

Body donation programs extend beyond logistics: they act as population-level health education and promotion initiatives, normalizing dialogue on death, solidarity, and legacy. Embedding integrative behavioral models into program design can strengthen institutional trust, support families, and ensure the sustainability of body donation worldwide.

## 1 Introduction

Body donation to science represents a personal and altruistic act in which an individual bequeaths their body to a university center to contribute to medical education and scientific research (1, 2, 41). This practice, which is vital for the training of healthcare professionals and biomedical advancements, occurs within a context of growing demand for human cadavers, driven by the increasing number of medical students and medical faculties worldwide (3, 4). However, the shortage of available bodies constitutes a major challenge, exacerbated by cultural, ethical, and legal factors (5–8).

The decision to donate one’s body is based on various motivations, including generosity, the desire to contribute to the advancement of scientific knowledge, and, at times, pragmatism concerning the costs or procedures related to traditional funeral arrangements (9, 10). However, the motivations of donors and the expectations of their families, which are often underexplored, are key factors in optimizing donation programs and communication strategies (11, 12). The sociocultural context and perceptions surrounding this practice vary by country, thus influencing both the profile of donors and the success of body donation initiatives (2, 10, 12, 41).

In Belgium, body donation practices are shaped by linguistic and cultural diversity, with regional variations in communication and public awareness. At the University of Liège, located in the French-speaking part of the country, the body donation program has experienced specific challenges and evolutions in recent years. Over the last decade, inquiries, pledges, and actual donations have fluctuated markedly, with 2020 representing a turning point coinciding with the COVID-19 pandemic. Inquiries declined from 382 (2014) to 104 (2020), pledges from 290 (2014) to 72 (2020), and actual donations from 94 (2015/2019) to 22 (2020). A gradual recovery followed, although none of these indicators had returned to pre-2020 levels by 2024 (Inquiries: ~240; Promises of Body Donation: ~140; Bodies Received: ~80). These dynamics provide the institutional and societal backdrop of the present exploratory study (13).

Despite the importance of understanding donation within this context, the voices of donors and families remain underrepresented in the literature. Family members, in particular, may experience what has been described as a “double loss”: first through the death of their loved one, and second through the temporary absence of the body during its use in teaching (14). Integrating their experiences into program development is essential not only for institutional trust but also for ensuring that body donation programs meet wider public health goals of transparency, equity, and social legitimacy.

The present study therefore aims to explore donor motivations and family perspectives in a Belgian university program through an exploratory service evaluation. By drawing on the experiences of individuals who have formally registered as body donors, as well as those of the relatives involved in this process, the study offers an initial understanding of the population engaging with the University of Liège Body Donation Program. By combining descriptive statistics with narrative insights, it seeks to highlight the human and ethical dimensions of body donation, and to generate lessons for both medical education and institutional practice.

To address these gaps, this study was guided by the following objectives:

(1) to examine the motivations of registered body donors at the University of Liège;
(2) to describe and analyze the experiences of donor families;
(3) to interpret these findings through integrative behavioral frameworks (BCW, PMT, prosociality) to inform institutional and public health practice.

## 2 Materials and methods

### 2.1 Study design

We conducted a mixed-methods exploratory study combining quantitative descriptive analyses and qualitative thematic analyses of open-ended responses. According to established classifications of mixed-methods research (15), this approach corresponds to a convergent parallel design, in which quantitative and qualitative data are collected during the same phase, analyzed separately, and integrated during interpretation to provide a comprehensive understanding of the phenomenon. The study was carried out at the University of Liège (ULiège) Body Donation Centre as part of an exploratory service evaluation aimed at informing institutional practice and medical education.

### 2.2 Populations and recruitment

Two populations were targeted:

- *Donors.* From 170 individuals who had pledged body donation in 2018, all were invited to participate. Inclusion criteria were age ≥ 18 years, proficiency in written French, informed consent, and questionnaire return before 31 March 2021. A total of 104 questionnaires were returned (response rate: 61.2%).
- *Families.* Relatives of deceased donors were contacted through funeral services between December 2020 and March 2021. Of 45 questionnaires distributed, 10 were completed and returned (22.2%).

As the ULiège program operates in French-speaking Wallonia, questionnaires were administered in French only.

Inferential statistics were applied exclusively to donor data; the small family sample was analyzed descriptively.

### 2.3 Instruments and validation

Two questionnaires were specifically developed:

- the *donor questionnaire* (17 items, 4 sections), which explored sociodemographic data, access to information, motivations, expectations, and advice for public awareness (Table 1);
- the *family questionnaire* (14 items, 3 sections), which addressed awareness of the donor’s decision, experiences of bereavement, and satisfaction with the donation program (Table 2).

**Table 1:** Questionnaire for body donors—structure and items.

Section	Item	Question/variable	Response options
A. Sociodemographic data	1	Age	___ years
	2	Place of birth (City/Country)	(Free text)
	3	Gender	□ Female □ Male □ Other
	4	Place of residence	(Free text)
	5	Occupation (before retirement)	(Free text)
	6	Education level	□ Primary □ Secondary □ Higher nonuniversity □ Higher university □ Other: ______
	7	Nationality	(Free text)
B. Access to information channels	8	How did you learn about body donation to science?	□ Radio □ Poster campaign □ Television □ University of Liège website □ Print media □ Word of mouth (Family/Friends/Colleagues) □ Other: ______
	9	Did you easily find the information about body donation to science?	□ Yes □ No – Please specify: ______
C. Motivations	11	What motivated you to donate your body to science?	(Free text)
	12	Have you discussed your decision to donate your body to science with your relatives/family?	□ Yes □ No
	13	What were their reactions?	(Free text)
	14	Why did you choose the University of Liège?	(Free text)
	15	Do you have specific expectations from our service regarding your family or relatives?	(Free text)
	16	Has your family influenced your decision to donate your body to science?	□ Yes – Please specify: ______ □ No – Please specify: ______
D. Practical advice	17	In your opinion, how should the population be made more aware of body donation to science?	(Free text)

**Table 2:** Questionnaire for families of deceased body donors—structure and items.

Section	Item	Question/variable	Response options
A. Awareness of the donor’s decision	1	Were you aware of your relative’s decision to donate their body to science?	□ Yes □ No
	2	Did you know their motivations?	(Free text)
	3	Do you think the donor’s motivations were respected?	(Free text)
B. Family cohesion	4	How did you experience this bereavement?	(Free text)
	5	Do you think the grieving process is different when body donation to science is involved?	(Free text)
	6	Is there a way to make this grieving process easier?	(Free text)
	7	How much time elapsed between the death and the restitution of the donor’s body?	(Free text)
	8	Was this waiting period reasonable?	□ Yes □ No – Please specify: ______
C. Satisfaction with the University of Liège Body Donation Centre	9	Were you informed about the time of the body’s restitution?	□ Yes □ No – If no, would you have wanted to be informed? □ Yes □ No
	10	What type of funeral was carried out?	□ Cremation □ Burial □ Unknown □ Other: ______
	11	Were you satisfied with the body donation service?	□ Yes □ No – Please specify: ______
	12	What advice would you give to improve our services?	(Free text)
	13	What is your relationship to the deceased?	(Free text)
	14	Did you complete this questionnaire alone?	□ Yes □ No – With whom? ______

Both instruments were designed with reference to the *Behavior Change Wheel (BCW)* and its COM-B model (capability, opportunity, motivation) (16). Items were mapped conceptually to these dimensions (Annex A).

Content validity was reviewed by a panel of 17 experts (four donation specialists, ten healthcare professionals, and three potential donors, including one with prior family experience). Face validity was assessed during a pilot test with three potential donors. Readability was optimized (large font, clear layout) given the advanced age of many donors. Because the questionnaires consisted mainly of categorical and open-ended items, internal consistency measures such as Cronbach’s alpha were not applicable. Instrument validity was ensured through expert review, piloting, and conceptual mapping to the BCW framework.

### 2.4 Data collection and management

Donor questionnaires were mailed with one postal reminder; family questionnaires were distributed via funeral services. Participation was voluntary and without compensation. Responses were anonymized, coded according to a predefined codebook, and securely stored. Identifiers and paper forms were destroyed after data entry.

### 2.5 Ethical considerations

The study *“Evaluation of the motivations and expectations of body donors and their families in the context of body donation to science at the University of Liège”* was reviewed by the Hospital-Faculty Ethics Committee of the University of Liège (ref. 2020/393). The committee confirmed that it did not fall under the Belgian law of 7 May 2004 on experiments involving human subjects. All participants provided written informed consent. Data handling complied with the General Data Protection Regulation (EU 2016/679).

### 2.6 Quantitative analysis

Categorical variables are presented as absolute numbers and percentages; continuous variables as medians with interquartile ranges (IQR). Normality of distributions was assessed by visual inspection and the Shapiro–Wilk test. Group comparisons used the Kruskal–Wallis test for continuous variables, while chi-square tests were applied for categorical variables with sufficient expected frequencies; Fisher’s exact test was used when expected cell counts were small. Statistical significance was set at *p* < 0.05. Inferential analyses were limited to donor data; family responses were described qualitatively and quantitatively without inferential testing.

### 2.7 Qualitative analysis

Open-text responses were analyzed using *thematic content analysis*. A deductive coding frame based on the COM-B model was refined inductively to capture emergent themes. Two coders independently analyzed a subset of responses; discrepancies were resolved by consensus. *Illustrative quotations are reported to foreground participants’ voices and provide context for numerical findings.*

## 3 Results

In line with the study objectives, the results are presented in two parts: (1) donor motivations, characteristics, and decision-making processes; and (2) experiences and perspectives of bereaved donor families. Quantitative and qualitative data are reported together when appropriate to enhance interpretation.

### 3.1 Quantitative analysis of donor responses

#### 3.1.1 Study population

Of the 170 individuals registered as donors in 2018, 104 returned complete questionnaires (response rate: 61.2%). Exclusions were due to non-response (*n* = 62), explicit refusal (*n* = 2), death prior to receipt (*n* = 1), or incomplete data (*n* = 1).

#### 3.1.2 Sociodemographic characteristics

The donor sample comprised 66 women (63.5%) and 38 men (36.5%), with a mean age of 72 years. Most participants were aged 60–79 years (*n* = 72, 69.2%). Almost all were Belgian nationals (*n* = 100, 96.2%), and the majority lived in Liège Province, where the university is located (*n* = 82, 78.9%). Regarding education, 43 donors (41.3%) had completed secondary studies and 36 (34.6%) non-university higher education. Most were employees or civil servants (*n* = 58, 55.8%). Links with medical/paramedical professions were reported by 12 donors (11.5%) and with education by 20 donors (19.2%) (Table 3).

**Table 3:** Sociodemographic characteristics of donors at the ULiège Body Donation Centre (*n* = 104).

Sociodémographic variables		
		*n* (%)
Sex	Male	38 (36.54)
	Female	66 (63.46)
Age	20–39 years	1 (0.96)
	40–59 years	12 (11.54)
	60–79 years	72 (69.23)
	≥ 80 years	19 (18.27)
Nationality	Belgian	100 (96.15)
	Other	4 (3.85)
Place of birth	Liège Province	62 (59.62)
	Belgium (other provinces)	29 (27.88)
	Abroad	13 (12.50)
Place of residence	Liège Province	82 (78.85)
	Belgium (other provinces)	22(2.15)
	Abroad	0 (0)
Educational level	Primary	10 (9.62)
	Secondary	43 (41.35)
	Higher nonuniversity	36 (34.62)
	University	14 (13.46)
	NA	1 (0.96)
Occupation	Self-employed	17 (16.35)
	Employee/Civil servant	58 (55.77)
	Worker/Technician	21 (20.19)
	Unemployed	8 (7.69)
Profession related to medical/educational fields	Medical/Paramedical	12 (11.54)
	Teacher	20 (19.23)
	Nonmedical	72 (69.23)

#### 3.1.3 Access to information about body donation

Word-of-mouth was the most frequent source of information (*n* = 55, 52.9%). Other sources included traditional or digital media (*n* = 15, 14.4%) and healthcare or teaching staff (*n* = 10, 9.6%). Twenty-four donors (23.1%) reported multiple sources. Almost all respondents (*n* = 100, 96.2%) found information easy to access.

When seeking additional information, donors most often turned to relatives (*n* = 26, 25.0%), the ULiège Body Donation Centre (*n* = 20, 19.2%), or physicians (*n* = 11, 10.6%). Preferred awareness strategies included traditional media and testimonials (*n* = 50, 48.1%), institutional or community relays (*n* = 17, 16.4%), and social media (*n* = 7, 6.7%) (Table 4).

**Table 4:** Information channels used by donors.

Information	
	*n* (%)
How did you first hear about body donation to science?	
Radio/TV, print media, awareness campaign, or website	15 (14.4)
From healthcare or teaching staff	10 (9.6)
Word of mouth	55 (52.9)
Multiple sources	24 (23.1)
Did you find information about body donation easily?	
Yes	100 (96.15)
No	4 (3.85)
Additional sources of information	
Internet search	5 (4.81)
Contact with the ULiège Body Donation Centre	20 (19.23)
Leaflet from the ULiège Body Donation Centre	2 (1.92)
Physicians	11 (10.58)
Funeral directors	2 (1.92)
Municipal administrations	3 (2.88)
Based on a relative’s choice or family tradition	5 (4.81)
Advice from relatives	26 (25)
Multiple sources	3 (2.88)
No response	27 (25.96)
According to you, how should the population be made aware of body donation to science?	
Traditional media (Radio/TV/Print/Internet/Advertising/Testimonials)	50 (48.08)
Social media (Facebook/YouTube/Instagram)	7 (6.73)
Poster campaigns/leaflets	3 (2.88)
Word of mouth (Hospitals/Physicians/Mutual insurance/Cultural venues/Schools/Municipalities/Relatives)	17 (16.35)
Financial compensation	3 (2.88)
Solidarity message (“love and support for those left behind”)	4 (3.85)
No response	20 (19.23)

#### 3.1.4 Communication of the decision to relatives and family reactions

Almost all donors (*n* = 100, 96.2%) had discussed their decision with relatives. Reported family reactions were mainly favorable (*n* = 66, 63.5%), followed by astonishment (*n* = 22, 21.2%) or sadness/mixed feelings (*n* = 16, 15.4%).

Explanations included respect for the donor’s choice (*n* = 26, 25.0%), affirmation of personal autonomy (*n* = 9, 8.7%), family agreement conditional on reflection or children’s approval (*n* = 4, 3.9%), and previous family practice of donation (*n* = 2, 1.9%). Concerns about grieving were reported in 15 donors’ families (14.4%) (Table 5).

**Table 5:** Donor communication with family regarding body donation decisions (*n* = 104).

Communication with family	
	*n*(%)
Have you discussed your decision to donate your body to science with your relatives/family?	
Yes	100 (96.15)
No	4 (3.85)
What were their reactions?	
Supportive	66 (63.46)
Surprised/Astonished	22 (21.15)
No reaction	0 (0)
Sadness or mixed feelings	16 (15.38)
Reasons for family reaction	
Donor’s decision (“my body/my choice”)	9 (8.65)
Family agreement after reflection/approval of children desired	4 (3.85)
Family respects my choice	26 (25)
Family habit (they will also donate)	2 (1.92)
Family concern about managing grief, waiting time, inability to gather, or fear of the unknown	15 (14.42)
Surprise	11 (10.58)
Absence of close relatives/family	2 (1.92)
No response	35 (33.65)

#### 3.1.5 Motivations, expectations, family influence, and choice of institution

The most frequent motivation was the desire to contribute to science (*n* = 59, 56.7%). Other motivations included symbolic meaning (*n* = 17, 16.4%), altruism (*n* = 13, 12.5%), gratitude for medical care (*n* = 11, 10.6%), and family-related reasons (*n* = 3, 2.9%). One donor (1.0%) did not specify a motivation.

Sixty-six donors (63.5%) expressed no specific expectations toward the institution. Among those who did, 25 (24.0%) asked for compassion toward families, six (5.8%) emphasized respect for their wishes, and two (1.9%) mentioned financial compensation.

Most donors (*n* = 92, 88.5%) declared their decision was not influenced by their family, while 12 (11.5%) reported some influence. Among the latter, explanations included respect for autonomy (*n* = 13, 12.5%) and family tradition of donation (*n* = 5, 4.8%).

The reasons for choosing the University of Liège were related primarily to geographical proximity (50%), followed by the institution’s reputation or alumni connection (19.23%), recognition of the University Hospital (20.19%), and, more rarely, attachment to the city of birth (4.81%) (Table 6).

**Table 6:** Donors’ motivations for body donation to science at the University of Liège (*n* = 104).

Motivation	
	*n*(%)
What motivated you to donate your body to science?	
Altruism	13 (12.5)
Desire to contribute to science (research/training of future physicians and surgeons)	59 (56.73)
Giving meaning to death	17 (16.35)
Gratitude for medical care received	11 (10.58)
Family reasons (absence of relatives/family tradition)	3 (2.88)
No response	1 (0.96)
Do you have specific expectations from our service regarding your relatives/family?	
No expectations	66 (63.46)
Respect for the body	1 (0.96)
Compassion and support for families	25 (24.04)
Respect for the donor’s wishes	6 (5.77)
Financial compensation	2 (1.92)
No response	4 (3.85)
Has your family influenced your decision to donate your body to science?	
Yes	12 (11.54)
No	92 (88.46)
Reasons for family influence on donation decision	
Personal decision (“my body/my choice”)	50 (48.08)
Family agreement after reflection/approval of children desired	3 (2.88)
Family respects my choice	13 (12.50)
Family tradition (they will also donate)	5 (4.81)
Family concern about grief, waiting period, inability to gather, or fear of the unknown	2 (1.92)
Surprise	1 (0.96)
Absence of close relatives/family	6 (5.77)
No response	24 (23.08)
Why did you choose the University of Liège?	
Proximity	52 (50)
Reputation of ULiège, link with alma mater	20 (19.23)
Recognition of the University Hospital	21 (20.19)
Attachment to city of birth	5 (4.81)
Only institution known	6 (5.77)

#### 3.1.6 Determinants of body donation

Primary motivations for donation did not differ significantly (all *p* > 0.05) according to age, sex, occupation, medical background, or education.

However, several associations emerged:

- *Choice of institution.* Reasons for choosing ULiège were significantly associated with profession (*p* = 0.012), education level (*p* = 0.012), and primary donation motivation (*p* = 0.004). Health professionals and university graduates more often cited the reputation of the university or its hospital, whereas participants with primary/secondary education more frequently mentioned geographical proximity. Donors motivated by gratitude toward medical care were also more likely to highlight institutional recognition compared with those motivated by altruism or symbolic meaning.
- *Family reactions.* Reported family reactions were associated with occupational category (*p* = 0.020). Employees and civil servants more frequently reported supportive reactions, whereas workers and technicians more often described sadness, ambivalence, or absence of clear reaction.

No significant associations were found between sociodemographic characteristics and (i) information channels used, (ii) ease of access to information, (iii) having discussed the decision with relatives, or (iv) the degree of family influence on the decision (all *p* > 0.05).

### 3.2 Analysis of bereaved donor families’ responses

This subanalysis focused on bereaved families of body donors whose remains were used for anatomical teaching at the University of Liège and subsequently returned to relatives via funeral services. Among 45 families contacted, 10 returned complete questionnaires (response rate: 22.2%). Respondents were mainly children (*n* = 6) and spouses (*n* = 3), with one joint spouse–child response. In most cases (90%), questionnaires were completed individually.

#### 3.2.1 Awareness of the donation decision

All families (*n* = 10, 100%) reported being informed of the donor’s decision. In every case, the primary motivation was identified as contributing to science, particularly research and the training of healthcare professionals. Narratives frequently linked this motivation to prior personal or professional experiences with illness or healthcare. Eight families (80%) felt the donor’s wishes had been fully respected, whereas two (20%) expressed uncertainty due to lack of detailed knowledge about how remains were used.

#### 3.2.2 Bereavement experience

Most families (*n* = 7, 70%) described bereavement as particularly challenging, often characterized as a “double loss”—first at the time of death and again at the return of the remains. Two families (20%) reported coping without difficulty, citing long-term preparation, and one (10%) found comfort in honoring the donor’s wishes.

Families suggested several improvements to ease bereavement: reducing the delay between death and restitution (*n* = 2, 20%), providing better informational and emotional support (*n* = 2, 20%), notifying families 1 month in advance of the return (*n* = 1, 10%), offering reassurance about respectful handling of the body (*n* = 1, 10%), ensuring faculty presence at restitution (*n* = 1, 10%), and formal expressions of gratitude (*n* = 1, 10%). Two families (20%) indicated that no further support was needed.

#### 3.2.3 Interval between death and return of remains

At ULiège, restitution delays range from 3 months to 3 years. In this cohort, six families (60%) reported a delay of ≤6 months, three (30%) between 7–12 months, and one (10%) > 12 months. Seven families (70%) considered this delay reasonable. All families were informed of the return date. Cremation was the most common funeral practice (*n* = 6, 60%), followed by burial (*n* = 3, 30%).

#### 3.2.4 Satisfaction with the body donation program

Satisfaction was unanimous (*n* = 10, 100%). Families valued honoring the donor’s wishes (*n* = 4, 40%), contributing to science (*n* = 4, 40%), and setting an example for others (*n* = 2, 20%). No dissatisfaction was reported. Suggested improvements included advance notification of restitution (*n* = 3, 30%), direct faculty contact with families (*n* = 2, 20%), and broader public awareness of the value of body donation (*n* = 1, 10%). Three families (30%) reported that no changes were needed, while one (10%) suggested consolidating the grieving process into a single phase to avoid the emotional strain of “double bereavement.”

#### 3.2.5 Thematic synthesis

Qualitative analysis of open-text responses identified three domains (Table 7):

*Communication and understanding.* Families consistently described donation as motivated by service to science, often grounded in health-related experiences.
*Impact on grieving.* Many highlighted emotional strain linked to a perceived two-stage bereavement, while others described resilience facilitated by preparation or alignment with donor values.
*Satisfaction and program enhancement.* Despite unanimous satisfaction, families suggested refinements to strengthen communication, provide earlier notification, reassure ethical respect for remains, and acknowledge donation through formal recognition.

**Table 7:** Summary of survey responses from bereaved donor families, combining quantitative data and expanded representative quotations.

Domain	Quantitative findings	Expanded representative qualitative quotes
Awareness of the donation decision	100% informed; 100% identified main motivation as contributing to science; 80% felt wishes were respected; 20% unsure	“Since always, it was his wish to donate his body to science.”/“To advance medicine.”/“To advance science — as a nurse, she was oriented toward helping others; she had two disabled grandchildren and hoped science could help them and others.”/“He suffered from severe scoliosis and hoped to contribute to understanding and curing this condition.”/“We hope the donation was useful; we trust it was.”
Bereavement experience	70% reported “double loss”; 20% no difficulty; 10% comforted by donor’s will	“It felt like losing him twice, even if we knew it was coming.”/“A double bereavement — the return was a shock, and everything had to start over psychologically.”/“We were prepared for over 10 years and there was no hesitation at the time of death.”/“Without difficulty, as she had often spoken of this wish, although medical staff at the ICU seemed unfamiliar with the process.”
Suggestions to ease bereavement	Reduce delay (20%); better info/support (20%); notify 1 month prior (10%); reassure ethical respect (10%); faculty contact at return (10%); formal recognition (10%); no help needed (20%)	“Reduce the time between removal and return of the body — is it materially possible for the University?”/“Provide more information for the children; for the spouse, everything was clear.”/“Reassure us about the respect given to the body throughout the process.”/“Support the mental strength of the surviving spouse.”/“Contact families personally to explain how things happen — it would feel less anonymous.”/“Acknowledge families with an official thank-you.”
Interval between death and return	≤ 6 months: 60%; 7–12 months: 30%; > 12 months: 10%; 70% considered reasonable	–
Funeral type	Cremation: 60%; Burial: 30%	–
Satisfaction with the program	100% satisfied; reasons: honoring donor’s will (40%), contributing to science (40%), exemplary gesture (20%)	“It was her choice, and I respected it.”/“It was my mother’s will, and I honored it.”/“For science to progress in fighting cancer.”/“I hope this donation will help students understand the human body better.”/“Absolutely — I have personally chosen to do the same.”
Suggestions for improvement	Advance notification (30%); faculty contact at return (20%); public awareness (10%); no change (30%); single-phase grief (10%)	“Notify us a month before the funeral date.”/“Increase public and hospital awareness of the importance of body donation for medical training.”/“Have a faculty representative present before the departure of the deceased.”/“Avoid making families grieve twice — in France, the body does not return, so grieving starts immediately.”

## 4 Discussion

Optimizing body donation programs requires more than operational efficiency; it demands a systemic understanding of the psychological, social, and cultural determinants shaping donor decisions and family experiences. As emphasized by Bagian et al. (17), institutional performance depends as much on relational and communicational dynamics as on administrative procedures. Our findings, interpreted through the Behavior Change Wheel (BCW), highlight key levers to strengthen trust, enhance engagement, and ensure the sustainability of donation programs. Four main themes emerge: (i) the hierarchy of motivations for donation, (ii) the autonomy of decision-making, (iii) the role of information channels, and (iv) the family experience of donation.

### 4.1 Scientific utility as the dominant motivation

Consistent with international literature, our study confirms that the leading motivation for body donation is the desire to contribute to science and education (56.7%), followed by symbolic meaning, altruism, and gratitude. Previous work has highlighted this primacy of scientific utility (9, 10, 12) while also emphasizing the multifactorial nature of motivations, where altruism, gratitude, and family traditions may coexist. Interestingly, pragmatic considerations such as reducing funeral costs—frequent in other contexts (12)—were nearly absent in our sample.

This suggests that body donation is not reducible to a single driver but emerges from a multidimensional reflection that combines scientific contribution with personal and existential values. While organ donation is often framed around altruism and duty (18, 19), whole-body donation appears to be a more reflexive act, tied to educational and symbolic significance. Our findings therefore enrich the literature by clarifying the relative hierarchy of these drivers in a Belgian cohort.

### 4.2 Decision-making autonomy embedded in social contexts

Nearly nine out of ten donors reported making their decision independently, with limited direct family influence. However, most had discussed their choice with relatives, whose reactions varied by occupational category: employees/civil servants more often expressed support, whereas workers/technicians more frequently reported sadness or ambivalence. These findings highlight what Sanner (20, 21) termed conditional autonomy: while the decision is framed as “my body, my choice,” its reception and legitimacy remain socially situated.

International studies converge on this interpretation: motivations are primarily personal (9, 10), yet family dialog plays a key role in legitimizing the choice and facilitating grief (7, 12). Cultural and religious frameworks also condition this autonomy, as seen in studies across Europe, South Africa, and Asia (5, 22, 23). Our data thus support the idea that body donation is both a profoundly individual decision and a relational act negotiated within family and cultural networks.

### 4.3 Word-of-mouth as the main communication channel

More than half of our donors first heard of body donation through word-of-mouth, underlining the central role of interpersonal trust in initial engagement. Yet nearly half also called for broader media campaigns to ensure wider public visibility. This duality reflects the complementarity of communication channels observed elsewhere: interpersonal networks provide credibility, whereas mass media and social platforms expand reach (24–26).

Anatomy educators and students also emerge as powerful intermediaries. Nearly one-third of our donors had professional ties to healthcare or education, which positions them as credible ambassadors of donation. Prior studies confirm that students and educators serve as primary messengers for donation values (27, 28). However, exposure to dissection can generate ambivalence (29, 30), underscoring the need for pedagogical framing and symbolic rituals. Gratitude ceremonies, as described by Guo et al. (31), play a critical role in embedding respect and humanism within anatomy education and extending these values to society.

### 4.4 Family experiences: satisfaction and the challenge of “double bereavement”

All families in our study reported satisfaction with the program and emphasized respect for donor wishes and scientific contribution. Yet many described the grieving process as fragmented, marked by a perceived “double bereavement”—at the time of death and later at the restitution of remains. This echoes thanatology research showing that disrupted rituals and delayed closure can complicate mourning (14, 32).

At the same time, some families reported that donation provided comfort, transforming loss into a meaningful contribution to society. This ambivalence, also observed in prior studies (33, 34), highlights the importance of tailored family support. Suggestions emerging from our cohort—timely notifications, reassurance about respectful handling, and faculty presence at restitution—mirror recommendations in the international literature for strengthening transparency, recognition, and ritual support (10, 31).

### 4.5 Toward an integrative model: protection motivation theory and prosociality

Beyond the BCW/COM-B framework, our findings can be interpreted through an integrative model combining Protection Motivation Theory (PMT) and prosocial and altruistic processes. PMT posits that behavior results from both a threat appraisal and a coping appraisal (35–37). In the context of body donation, the threat dimension takes two forms: a systemic threat (the shortage of bodies compromising the quality of medical training, as reported by a majority of donors) and an existential threat (awareness of mortality and the search for meaning at the end of life).

On the coping appraisal side, our data indicate that:

- Perceived response efficacy is central, with more than half of respondents ranking scientific and educational contribution as the primary motivation (56.7%).
- Self-efficacy is strengthened by the accessibility of information and by the fact that nearly all donors informed their families (96.2%), reflecting a sense of control over the decision.
- Perceived costs are mainly emotional and ritual in nature, expressed by families through the experience of a “double bereavement” (at the time of death and at the restitution of the body), and modulated by family reactions that varied according to socio-professional background.

Taken together, these elements delineate a protective motivation, whereby body donation is construed as both a response to a collective threat (insufficient resources for medical education) and a way of attributing meaning to one’s own finitude.

In parallel, prosocial and altruistic drivers illuminate other dimensions of our results: empathy toward future generations, social responsibility, gratitude toward caregivers, and the *warm-glow* of a valued contribution. This interpretation is consistent with the literature on costly prosocial behaviors (38, 39) and supports the view that body donation is embedded in a public good logic. Our data confirm this perspective: although explicit altruism was not the most frequently cited motivation, it underlies the observed motivational hierarchy and emerges in families’ suggestions for program improvement (rituals, institutional recognition).

**Figure 1:**
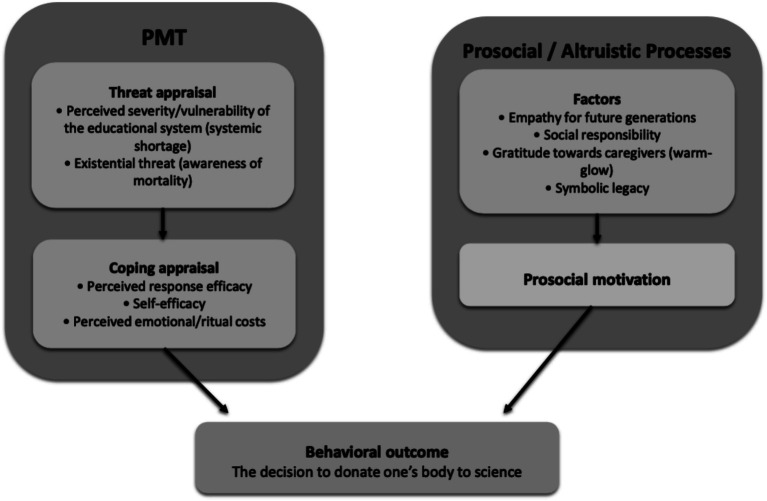
An integrative model combining Protection Motivation Theory (PMT) with prosocial and altruistic processes to explain the decision to donate one’s body to science. In the context of body donation, Protection Motivation Theory (PMT) explains the decision as a protective response to both the perceived shortage affecting medical education (systemic threat, perceived severity/vulnerability) and the existential fear of mortality (response efficacy, self-efficacy, perceived costs). In parallel, altruistic factors—including empathy for future generations, social responsibility, gratitude toward caregivers (warm-glow), and symbolic legacy—nurture a prosocial motivation. The integration of these two pathways demonstrates that body donation simultaneously fulfills a protective function and an altruistic contribution to the common good, ultimately leading to the behavioral outcome.

Thus, integrating PMT with prosociality provides a framework in which body donation simultaneously responds to a protective logic (mitigating a threatening shortage, making sense of mortality) and an altruistic logic (supporting student education, contributing to the common good). The almost systematic presence of family support can be understood as both a motivational amplifier and a perceived cost reducer, consistent with meta-analyses linking social support and prosocial behavior (40). This dual mechanism is illustrated in Figure 1, which conceptualizes body donation as the outcome of an interaction between protective and altruistic motivations.

### 4.6 Implications within the BCW framework

When transposed into the BCW/COM-B framework, this integrative model highlights several concrete levers for intervention:

- *Capability*: Clear and accessible information materials (brochures, videos, FAQs), combined with structured bereavement anticipation pathways (standardized timelines, designated faculty contact points), reduce uncertainty, enhance self-efficacy, and mitigate emotional costs.
- *Opportunity*: Expanding communication channels beyond word-of-mouth (community campaigns, hospital-based relays, trained educators), implementing standardized advance notices for body restitution, and ensuring faculty presence along with ceremonies of gratitude extend outreach, reinforce prosocial norms, and diminish ritual-related costs.
- *Motivation*: Testimonies from students and educators, concrete feedback on the pedagogical use of donations, structured scripts for family discussions, checklists, and visible forms of institutional recognition (letters, symbols, ceremonies) strengthen perceived response efficacy, sustain self-efficacy, and activate altruistic drivers such as the *warm-glow* effect or symbolic legacy.

These domains and their applications are summarized in Table 8, which integrates empirical findings with BCW-inspired interventions and specific targets linked to PMT and prosociality. Several of these initiatives are already being implemented at the University of Liège (awareness campaigns, community partnerships, ceremonies of gratitude). The analysis of trends from 2014 to 2024 suggests that such strategies have contributed to the post-pandemic recovery in pledges and actual body donations.

**Table 8:** Application of the COM-B framework to body donation: determinants, interventions, expected outcomes, and PMT/prosociality targets.

COM-B dimension (capability, opportunity, motivation)	Identified determinants	Examples of interventions	Expected outcomes	PMT/prosociality targets
Capability (knowledge and emotional resources)	Limited understanding of procedures; difficulty anticipating restitution; emotional vulnerability of families	Clear brochures/videos; transparent communication on pedagogical use; bereavement anticipation pathways (standardized timelines, designated contact points)	Improved comprehension; reduced uncertainty; better supported grieving process	↓ Perceived costs; ↑ Self-efficacy; stronger sense-making (social value)
Opportunity (social and environmental context)	Predominance of word-of-mouth; limited outreach; teachers as intermediaries	Media campaigns (TV/radio/press/web); community & hospital relays; teacher training; standardized restitution notice; faculty presence; ceremonies of gratitude	Broader audience; strengthened legitimacy; consolidated culture of respect; reduced ritual costs	↑ Perceived efficacy; ↑ Prosocial norms; ↓ Ritual/emotional costs (“double bereavement”)
Motivation (reflective and automatic)	Scientific utility as primary driver; altruism, gratitude, search for meaning; ambivalent family reactions	Student/teacher testimonials; concrete pedagogical use cases; structured family discussion scripts; checklists; ethical reassurance/traceability; formal recognition of families	Reinforced pride and legitimacy; symbolic transformation of loss; increased long-term adherence	↑ Response efficacy; ↑ Self-efficacy; warm-glow / recognition

### 4.7 Limitations of the study

This mixed-methods exploratory study has several limitations that must be considered when interpreting the findings.

First, the response rate among families of deceased donors was low (22.2%), which restricts the generalizability of results regarding bereavement and satisfaction. Families who chose to respond may represent those with particularly strong views, introducing potential self-selection bias. Silent or ambivalent perspectives are therefore likely underrepresented, a challenge frequently noted in research on end-of-life experiences (32, 33).

Second, the small family sample (*n* = 10) limits statistical power and precludes multivariable analysis. Findings from this group should thus be viewed as exploratory, highlighting themes that align with international literature—such as “double bereavement” and the importance of symbolic recognition—rather than establishing definitive trends.

Third, the cross-sectional design captures donor and family perspectives at a single point in time. It cannot account for how motivations, experiences, or satisfaction may evolve over the course of bereavement. Longitudinal studies would be necessary to explore these dynamics more fully.

Fourth, the study population was geographically concentrated in Liège and predominantly Belgian. Cultural, religious, and legal contexts vary widely across countries, and caution is needed when extrapolating these findings to other settings. Multicentric comparisons could help distinguish local from universal determinants of donation.

Fifth, the qualitative component relied exclusively on written responses to self-administered questionnaires. While this approach allowed for efficient and anonymous data collection on sensitive issues such as grief, ambivalence, and motivations, it inherently limited the depth, contextualization, and emotional nuance of participants’ accounts. Written responses tend to be more concise and less elaborated than data generated through interviews, which restricts the level of interpretive richness that can be achieved. Future studies incorporating interviews or focus groups would therefore provide a more detailed and dynamic understanding of donor motivations and family experiences.

Finally, while the self-administered questionnaires allowed for efficient data collection, they limited the depth of responses on sensitive issues such as grief or ambivalence. Complementary qualitative approaches, such as interviews or focus groups, could provide richer insights.

Despite these limitations, the study provides valuable empirical evidence in an underexplored field and offers practical directions for optimizing body donation programs.

### 4.8 Future research directions

This study opens several avenues for future research and program development. First, multicentric studies across different linguistic, cultural, and legal contexts could test the transferability of the integrative PMT–prosociality model and identify context-specific determinants of donation. Second, longitudinal designs following donors and families over time—from the pledge of donation to restitution and beyond—would clarify how motivations, expectations, and bereavement trajectories evolve and how institutional practices shape these trajectories. Third, in-depth qualitative work (e.g., semi-structured interviews or focus groups) with under-represented or non-responding families could capture silent forms of ambivalence, resistance, or mistrust that are less accessible through questionnaires. Finally, intervention studies evaluating BCW-informed strategies (communication tools, bereavement pathways, gratitude ceremonies, feedback to families) could generate robust evidence to guide anatomy departments, body donation centers, ethics committees, and public health authorities in designing ethically robust, family-sensitive, and sustainable donation pathways.

### 4.9 Conclusion

This exploratory study makes three principal contributions. *Empirically*, it provides rare and systematic data on donor motivations and family experiences within a European cohort. *Conceptually*, it advances an integrative model combining Protection Motivation Theory (PMT) and prosociality, framing body donation simultaneously as a response to existential threat and as an expression of social responsibility. *Operationally*, it translates these findings into actionable levers within the BCW/COM-B framework, proposing targeted interventions to enhance perceived efficacy, reduce emotional costs, and strengthen practices of recognition.

From a *public health perspective*, body donation programs emerge not only as institutional services but also as initiatives of education and health promotion at the population level, where discussions on death, solidarity, and legacy are normalized. Interventions that encourage family dialogue, foster transparency, and acknowledge donor contributions may broaden engagement while shielding vulnerable families from the experience of fragmented grief.

Although limited by sample size and geographical scope, the convergence with international literature suggests that the issues identified resonate beyond Belgium. More firmly embedding body donation within educational and public health agendas offers a promising avenue to reinforce institutional trust, support families, and ensure the sustainable growth of donation programs worldwide.

## Data Availability

The raw data supporting the conclusions of this article will be made available by the authors, without undue reservation.

## Annex A. Mapping of questionnaire items to COM-B dimensions

**Table:**

Questionnaire item	Target population	COM-B dimension	Rationale
Age, sex, education, occupation, place of residence	Donors	Capability (contextual)	Background factors shaping knowledge, literacy, and access to donation information.
How did you learn about body donation?	Donors	Opportunity	Reflects external sources of information and social exposure.
Did you find information easily?	Donors	Capability	Indicates perceived ability to access and understand relevant resources.
What motivated you to donate your body to science?	Donors	Motivation	Captures altruistic, symbolic, pragmatic, or gratitude-based determinants.
Have you discussed your decision with relatives?	Donors	Opportunity / Motivation	Social context and interpersonal validation of the decision.
What were their reactions?	Donors	Opportunity / Motivation	Influence of social environment on decision-making and persistence.
Why did you choose the University of Liège?	Donors	Opportunity	Institutional accessibility, proximity, or perceived reputation.
Do you have specific expectations for your family?	Donors	Motivation	Anticipated outcomes for relatives (compassion, respect, continuity).
Has your family influenced your decision?	Donors	Opportunity / Motivation	Family dynamics as social facilitators or barriers.
How should the population be made more aware?	Donors	Opportunity	Suggested strategies for enhancing societal awareness.
Were you aware of your relative’s decision?	Families	Opportunity	Transmission of the decision within the family context.
Did you know their motivations?	Families	Motivation	Understanding the symbolic or altruistic drivers of the donor.
Do you think the donor’s motivations were respected?	Families	Motivation	Evaluation of program alignment with donor’s wishes.
How did you experience this bereavement?	Families	Capability / Motivation	Personal coping ability and emotional response.
Do you think grieving is different with body donation?	Families	Motivation	Perception of specific emotional challenges tied to donation.
Is there a way to ease this grieving process?	Families	Opportunity	Suggestions for institutional or social support.
How much time elapsed between death and restitution?	Families	Opportunity	Structural/institutional factor affecting family experience.
Was this waiting period reasonable?	Families	Motivation	Evaluation of satisfaction and acceptability of program logistics.
Were you informed about the restitution?	Families	Opportunity	Communication and transparency from the institution.
What type of funeral was carried out?	Families	Opportunity	Reflects cultural and logistical continuity after donation.
Were you satisfied with the body donation service?	Families	Motivation	Overall evaluation of program quality and respect.
What advice would you give to improve our services?	Families	Opportunity / Motivation	Concrete proposals for change and expectations toward the institution.
